# Elucidating the expression and function of Numbl during cell adhesion-mediated drug resistance (CAM-DR) in multiple myeloma (MM)

**DOI:** 10.1186/s12885-019-6446-y

**Published:** 2019-12-30

**Authors:** Yuejiao Huang, Xianting Huang, Chun Cheng, Xiaohong Xu, Hong Liu, Xiaojing Yang, Li Yao, Zongmei Ding, Jie Tang, Song He, Yuchan Wang

**Affiliations:** 1 Department of Oncology, Nantong University Cancer Hospital, Nantong, Jiangsu 226001 People’s Republic of China; 2 Department of Oncology center, Jiangsu Jiangyin People’s Hospital, Jiangyin, Jiangsu 214400 People’s Republic of China; 3 Department of Pathogenic Biology, School of Medicine, Nantong University, Nantong, Jiangsu 226001 People’s Republic of China; 4 Department of Hematology, Affiliated Hospital of Nantong University, Nantong, Jiangsu 226001 People’s Republic of China; 5 Department of Immunology, Medical College of Jiangnan University, Wuxi, Jiangsu 214122 People’s Republic of China; 6 Department of Pathology, Nantong University Cancer Hospital, Nantong, Jiangsu 226001 People’s Republic of China

**Keywords:** MM, Numbl, Integrin β1, CAM-DR

## Abstract

**Background:**

Cell adhesion-mediated drug resistance (CAM-DR) is a major clinical problem that prevents successful treatment of multiple myeloma (MM). In particular, the expression levels of integrin β1 and its sub-cellular distribution (internalization and trafficking) are strongly associated with CAM-DR development.

**Methods:**

Development of an adhesion model of established MM cell lines and detection of Numbl and Integrinβ1 expression by Western Blot analysis. The interaction between Numbl and Integrinβ1 was assessed by a co-immunoprecipitation (CO-IP) method. Calcein AM assay was performed to investigate the levels of cell adhesion. Finally, the extent of CAM-DR in myeloma cells was measured using cell viability assay and flow cytometry analysis.

**Results:**

Our preliminary date suggest that Numbl is differentially expressed in a cell adhesion model of MM cell lines. In addition to binding to the phosphotyrosine-binding (PTB) domain, the carboxyl terminal of Numbl can also interact with integrin β1 to regulate the cell cycle by activating the pro-survival PI3K/AKT signaling pathway. This study intends to verify and elucidate the interaction between Numbl and integrin β1 and its functional outcome on CAM-DR. We have designed and developed a CAM-DR model using MM cells coated with either fibronectin or bone marrow stromal cells. We assessed whether Numbl influences cell-cycle progression and whether it, in turn, contributes to activation of PI3K/AKT signal pathway through the adjustment of its carboxyl end. Finally, we showed that the interaction of Numbl with integrin β1 promotes the formation of CAM-DR in MM cells.

**Conclusions:**

Our findings elucidated the specific molecular mechanisms of CAM-DR induction and confirmed that Numbl is crucial for the development of CAM-DR in MM cells.

## Background

Multiple myeloma (MM), also named as plasma cell myeloma, is a relatively indolent yet therapeutically challenging neoplasm, with a median survival period of 3 to 5 years [1]. Currently, treatment options are limited and include chemical drugs and biological target therapy. Given the high relapse rate and increased risk for drug resistance, there is a pressing need to identify potential therapeutic targets [2]. More specifically, the drug resistant nature of MM is the major obstacle for the inefficacy of chemotherapy. Previous studies have shown that the bone marrow microenvironment is the main factor influencing the resistance of MM cells [3]. This microenvironment-mediated resistance (EM-DR) consists of soluble factor-mediated drug resistance (SFM-DR) and cell adhesion-mediated drug resistance (CAM-DR), mainly involved in mediating the initial drug resistance. The initial drug resistance confers survival advantage to MM cells after preliminary drug exposure, leading to the formation of acquired drug resistance. Research suggests that CAM-DR is one of the major factors contributing to the development of drug resistance. A number of published studies confirm that MM cell adherence to stromal cells leads to most of the drug resistant phenotypes, and that disruption of signals mediating cell adhesion could significantly increase the sensitivity of tumor cells to chemotherapy drugs [4–6].

Studies have shown that members of the Integrin family of adhesion proteins are intimately involved in the mechanism of CAM-DR generation [2, 7, 8]. The signal cascade and cytoskeletal rearrangements induced by integrins are important etiological underpinnings of CAM-DR. Integrins are transmembrane glycoproteins made up of heterologous dimers and are localized at the cell surface. The integrins induce cell proliferation, retraction and migration. They also act as a adhesion receptors to link matrix proteins in between cells and facilitate the interaction between cells and the extracellular matrix (ECM). Integrins consist of 18 alpha subunits and 8 β subunits in total. These subunits form a total of 24 canonical integrins in different cell types [9]. Integrin β1 is one of the integrins that is widely distributed and has been studied extensively. The current consensus is that integrin β1 can combine with at least 12 different types of alpha subunits to coordinate the processes of cell adhesion and migration. Numerous studies have noted the high expression levels of integrin β1 in a variety of invasive cancers. Berry M,G [10], and other investigators have reported high expression of integrin β1 in highly invasive cancer cells isolated from breast cancer patients with low survival rates. This correlation suggested that the high expression of integrin β1 could be a robust candidate as biomarker for breast cancer. Moreover, curtailment of integrin β1 function by monoclonal antibodies has been shown to inhibit the invasiveness of breast cancer cells by approximately 80% [10]. Integrin β1 has also been reported to be highly expressed in patients with other malignancies, including gastric cancers, small cell lung cancers, and other tumors, suggestive of poor prognosis for patients [11, 12]. Recent studies have highlighted that integrin β1 mediated adhesion between cancer cells and the ECM can reduce the tumor cell cytotoxicity that is induced by radiotherapy and chemotherapy [13, 14]. Consistently, Naci D [15] and others have found that integrin β1 mediated adhesion between leukemic cells and the ECM can significantly inhibit the apoptosis induced by chemotherapy drugs. Further lending support to these findings, there is evidence to suggest that adhesion between mantle cell lymphoma (MCL) cells and the ECM is caused, in part, by abnormally elevated expression levels of integrin β1, an important factor of tumor cell resistance to therapy [16].

Integrin β1 is one of the major cell surface receptors mediating cell-cell and cell-extracellular matrix adhesion, which can affect tumor cell dynamics, including adhesion, migration, survival, and other biological processes [17]. Research suggests that the expression levels of integrin β1 is mainly regulated by its internalization cycle itself. Integrin β1 internalization cycle involves three major processes: 1) Endocytosis by plasma membrane, 2) Shuffling of integrin β1 to internal circulation particles, and 3) translocation back to the plasma membrane [18, 19]. In the whole process of internalization cycle, a considerable load of the internalized integrin β1 will be transferred to the intracellular polycystic particles and degraded in the lysosomes [20, 21]. The molecules that have, thus far, been found to be involved in the regulation of integrin β1 internalization cycle include: Syntaxin [18], plasmalemme neuraminidase (NEU3) [20], and the adaptin Numb [22]. Numb, also referred to as cell fate determinant, is a type of phospho-tyrosine binding (PTB) domain-containing protein connected to intracellular membranes. Numb is involved in many important physiological and pathological processes, such as cell differentiation, proliferation, apoptosis, regeneration, and tumorigenesis. Studies have shown that Numb interacts with integrin β1 through its PTB domain and affects the cell adhesion by dictating the position of integrin β1 internalization [23]. Numbl is the analogue of Numb homologue. It is a 609 amino acid-long protein and its expression exhibits high tissue specificity. Numbl is mainly distributed in the embryo, adult brain, nerve tissue, muscle cells and peripheral lymphocytes [24]. Numbl and Numb possess the same PTB domains. By using a two-hybrid yeast technique, Bogdanovic Ozren [25] and others have demonstrated that PTB domain of Numbl can also interact with integrin β1 and coordinate its internalization transfer process in the cells. This suggests that Numbl, in some contexts, functions in a manner similar to Numb. Additional studies have shown that PTB domain structure, combined with integrin β1, can contribute to the internalization and lysosomal degradation of integrin β1 [25].

Research into the underpinnings of CAM-DR revealed that when tumor cells adhere to the extracellular matrix or stromal cells, the cell cycle arrests in G0/G1 phase, thereby preventing tumor cells from completing the cell cycle. Such relatively quiescent state of tumor cells have been shown to neutralize the cytotoxic effects of drugs, leading to insensitivity or resistance towards chemotherapy drugs [7, 26, 27]. Studies have shown that cell cycle arrest is closely related to an increase in the protein levels of cell cycle negative regulatory factor p21^WAF/Cip^, p27^Kip1^ during the development of CAM-DR in tumor cells [28]. In the model of CAM-DR in mantle cell lymphoma, we found that when malignant cells adhered to the bone marrow stromal cell line, HS-5, the p27^Kip1^ ubiquitination degradation significantly reduced the accumulation of p27^Kip1^ and the cell cycle arrest was triggered by an increase in p27^Kip1^ protein levels [29]. Furthermore, intracellular accumulation of p27^Kip1^ induced by the binding between soluble extracellular matrix and cancer cell integrin receptors led to CAM-DR development [30]. Papers have also demonstrated that in addition to altering the cell cycle, integrin β1 can activate the downstream corresponding signal pathways during the process mediating cell adhesion. This includes focal adhesion kinase (FAK), integrin-link kinase, and phosphatidylinositol 3-kinase (PI3K) / protein kinase B (PKB/AKT) [31–33]. The activation of these processes synergistically lead to the development of tumor cell resistance to chemotherapeutic agents [34, 35].

In summary, Numbl plays a significant role in the formation of CAM-DR in MM cells. Based on previous work, we have drawn the following hypothesis: During the process of MM cell adhesion, distinct Numbl binding domains regulate spatio-temporal distribution and expression levels of integrin β1, resulting in arrest of cell cycle and triggering the corresponding signaling pathways that lead to the occurrence CAM-DR in MM cells.

## Methods

### Cell cultures

RPMI 8226 and H929 human multiple myeloma (MM) cell lines and HS-5 bone marrow stromal cell line were obtained from Chinese Academy of Sciences (Shanghai, China). RPMI 8226, and H929 MM cell lines and HS-5 cell line were cultured in RPMI 1640 medium (GibCo BRL, Grand Island, NY, USA) and in F12 medium (GibCo BRL, Grand Island, NY, USA) respectively. Each media were supplemented with 10% fetal bovine serum (FBS)(Hyclone, ThermoFisher Scientific, Waltham, MA, USA), 2 mM L-glutamine (GibCo BRL, Grand Island, NY, USA), and 100 U/mL penicillin–streptomycin mixture (GibCo BRL, Grand Island, NY, USA) at 37 °C and 5% CO2.

### Adhesion assays

RPMI 8226 and H929 cells adhesion assay was performed following instructions by Damiano et al. (1999) [36]. HS-5 cells were seeded at 1 × 106 cells/ml and incubated at 37 °C and 5% CO2 overnight before being washed 3X in serum-free RPMI1640 medium. RPMI 8226 and H929 cells were plated with HS-5 cells for 2 h in serum-free RPMI 1640 medium. Non-adhered cells were then removed but adhered cells were further incubated overnight in RPMI 1640 supplemented media.

To assess adhesion rate [37, 38], RPMI 8226 and H929 cells were labeled with 5 uM of Calcein-AM (Santa Cruz Biotechnology, CA, USA) for 30 mins, washed and incubated an additional 45 min to ensure that unbound dyes diffuse out of the cells. Next, labeled cells were washed 3X with phosphate-buffered saline (PBS) then incubated for 2 h. Finally, the absorbance was read out by Multiskan MK3 (ThermoFisher Scientific, USA) at the wavelength of 490 nm for 3X.

### Western blot analysis

To measure the relative preponderance of proteins of interest using Western Blot (WB), we followed an optimized protocol. Cell lysates were homogenized in lysis buffer (50 mM Tris–HCl (pH 7.4), 120 mM NaCl, 0.5% Nonidet P-40, 100 mM NaF, 200 M Na3VO4, and protease inhibitor mixture), then centrifuged at 8000–10,000 rpm for 30 min to collect the supernatant at 4 °C. The whole-cell lysates were subjected to 10–12% gradient polyacrylamide gels and transferred to Polyvinylidene Fluoride (PVDF) membrane (03010040001, Rocho, UK). After blocking with 5% nonfat milk for 1 h at room temperature, the primary antibodies were incubated overnight at 4 °C. After 3 times washes (5 min/wash) in PBST (PBS containing 0.1% Tween-20), the membrane was then incubated with HRP-labeled secondary antibody for 2 h at room temperature about 25 °C. Then the enhanced chemiluminescent (ECL) detection systems developed the membrane. All the antibodies utilized in this study with the details as follows: anti-Numbl (anti-rabbit, 1:1000, Sigma-Aldrich), anti-Integrin β1 (anti-mouse, 1:1000, Sigma-Aldrich), anti-P27kip1 (anti-rabbit, 1:500, Santa Cruz Biotechnology), anti-CDK2 (anti-rabbit, 1:500, Santa Cruz Biotechnology), anti-GFP (anti-rabbit, 1:500, Santa Cruz Biotechnology), anti-HA (anti-mouse, 1:500, Santa Cruz Biotechnology), cleaved-caspase 3 (anti-rabbit, 1:500, Santa Cruz Biotechnology), anti-Bcl 2 (anti-mouse, 1:500, Santa Cruz Biotechnology), anti-GAPDH (anti-rabbit, 1:1000, Sigma), anti-AKT (anti-rabbit, 1:1000, Cell Signaling), anti-p-AKT (anti-rabbit, 1:1000, Cell Signaling), anti-FAK (anti-rabbit, 1:1000, Cell Signaling) and anti-p-FAK (anti-rabbit, 1:1000, Cell Signaling).

### Immunofluorescence staining

MM cells were collected and washed twice with -cold PBS, and incubated for 10 min at 4 °C in Triton X-100 lysis buffer (30 mM Tris-HCl pH 7.5, 150 mM NaCl, 25 mM NaF, 1% Triton X-100, 10% glycerol, 2 mM Naorthovanadate). MM Cells were immobilized for 20 min with cold PBS containing 4% formaldehyde (Sigma Aldrich), permeabilized with 0.1% Triton X-100 for 10 min, and then incubated for 2 h at 4 °C in 1% BSA. Then the cells were incubated with primary antibodies at 4 °C overnight. After washing in PBS, cells was incubated with appropriate sheep FITC- or TRITC- conjugated secondary antibodies (1:250; Jackson ImmunoResearch Laboratories) for 2 h at RT. Sections were washed in PBS and counterstained with DNA stains using 4′6-diamidino-2-phenylindole dihydrochloride (Dapi, Sigma). Finally, cells were reversed on glass slides with glycerol and PBS (1:1). Immunolabeled cells were examined under a Leica Confocal Laser Scanning Microscope and fluorescence microscope. The following antibodies were used for immunofluorescence: anti-integrin-β1 at 1:300 (MAB1981, Chemicon); anti-Numbl at 1:200 (ab37500, abcam).

### Plasmids and transient transfection

The full-length human Numbl (GenBank no. NM_004756.3) and human ITGB-1 (GenBank no. NM_001113770.1) cDNA were isolated from the cDNA library. For RNA interference (RNAi), sequences targeting the Numbl (AAGGCAAAGCCACTGTAGAGA) were cloned into the pCDNA6.2-GW/enhanced green fluorescent protein–micro RNA vector, as per the instruction manual (Invitrogen, Carlsbad, CA). Scrambled RNA oligonucleotides were used as controls. For each well, 33.3 nM of each of the three oligos were transfected using Lipofectamine 2000 (Invitrogen, Carlsbad, CA) according to the manufacturer’s instructions. The medium was replaced after 6 h of incubation with RPMI 1640 containing 10% FBS. After at least 48 h, transfected cells were used for the subsequent experiments.

### Co- immunoprecipitation and immunoblotting

MM cells were collected and lysed in RIPA buffer in ice for the immunoprecipitation assays. After preclearance with 20 μl protein G Sepharose beads (Roche, Basel, Switzerland) for 1 h at 4 °C, cell lysates were incubated with the anti-Numbl and anti-Integrin β1 bound to either protein A or G Sepharose beads rotating for 12 h at 4 °C. Then the precipitated immune complexes were washed with RIPA lysis buffer at least 4 times, eluted by boiling in 2 × SDS sample buffer, separated by SDS–PAGE gel, and analyzed using enhanced chemiluminescence system.

### Cell cycle analysis

To analyze cell cycle of MM cells, cells were first collected and fixed in 70% ethanol for 1 h at 4 °C, then incubated with RNase A (1 mg/mL, sigma, USA) for 30 min at 37 °C incubator. Subsequently, cells were stained with 50 mg/mL propidium iodide (PI) (Becton–Dickinson, San Jose, CA, USA) in PBS, 0.5% Tween-20, and analyzed using a Becton–Dickinson flow cytometer BD FACScan (Becton–Dickinson, USA).

### Flow cytometry-based Annexin V/PI staining

The flow cytometric analysis was performed to analyze the extent of both apoptosis and necrosis of MM cells using an ApoScreen Annexin V-FITC kit (Southern Biotechnology, Birmingham, AL, USA), according to the manufacturer’s protocol. In brief, RPMI 8226 and H929 cells were washed and resuspended in cold binding buffer [10 mM N-2-hydroxyethylpiperazine-N-ethane-sulphonicacid (HEPES), pH 7.4, 140 mM NaCl, 2.5 Mm CaCl2, 0.1% BSA] at cell concentrations between 1 × 106 and 1× 107 cells/ml. After washing, 10 μl labeled annexin V, 380 μl binding buffer and 10 μl PI solution were added to the cell suspension in sequence on ice. Subsequently, the number of stained cells was measured via flow cytometry BD FACScan (Becton Dickinson, San Jose, CA, USA) and analyzed by FlowJo 10.

### Drug cytotoxicity assay

For drug cytotoxicity assays, MM cells were washed once and adhered to FN or stromal cells, as previously described [39]. In our preliminary experiment [39], the proper drug concentration needed to induce apoptosis in our cell lines had already been established. The viability of MM cells following addition of Doxorubicin, Mitoxantrone or Dexamethasone, at varying concentrations (1 μM, 2 μM and 0.05 μM), for 72 h, was assessed using Cell Counting Kit-8 (CCK8) assay and trypan staining (*, *P* < 0.05 compared with the group of control). For drug exposure, after 24 h, drugs or vehicle control were added to each well and incubated for an additional 72 h, after which the medium containing drugs was removed and the suspended and attached MM cells were collected.

To evaluate cell viability, cells were seeded on a 96-well cell culture cluster (Corning Inc., Corning, NY) at a concentration of 5 × 10^4^/well in a volume of l L and were grown overnight. CCK8 reagents (Dojindo, Kumamoto, Japan) were added to the different subset wells and the cells were incubated at 37 °C and 5% CO_2_. The absorbance was quantified using an automated plate reader at a test wavelength of 450 nm for at least three times.

### Statistical analysis

All experiments were repeated at least three times. All numerical data are described as mean ± SD. Data was analyzed using the two-tailed *t* test. A *P* value < 0.05 was considered statistically significant in all of the analyses.

## Results

### The expression of Numbl during MM cell adhesion to HS-5 cells or FN

**Fig. 1:**
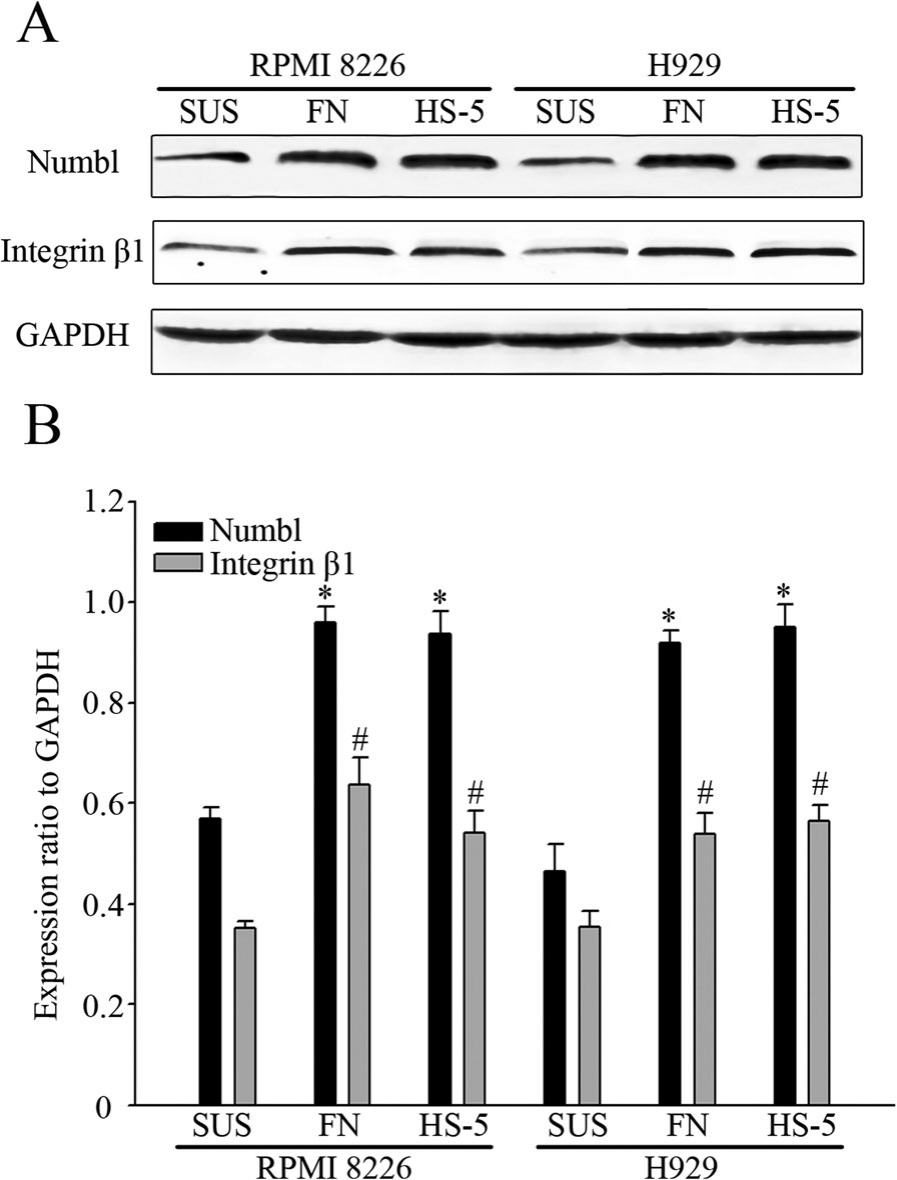
Expression of Numbl in myeloma cell in adherent co-culture and suspension. **a** Western Blot analysis detected the expression of Numbl and Integrin β1. **b** The gray value quantification of (**a**). *, # compared to suspension group (SUS), *P* < 0.05

Initially, we co-cultured two independent types of human multiple myeloma cell lines, namely, RPMI-8226 and H929, with either a bone stromal cell line, HS-5, or fibronectin (FN). An MM cell suspension culture was used as a control. As shown in Fig. 1, the expression of Numbl and Integrin β1 were increased when RPMI-8226 or H929 cells adhered to FN or HS-5 cells in co-cultures.

### Numbl interacts with integrin β1

**Fig. 2:**
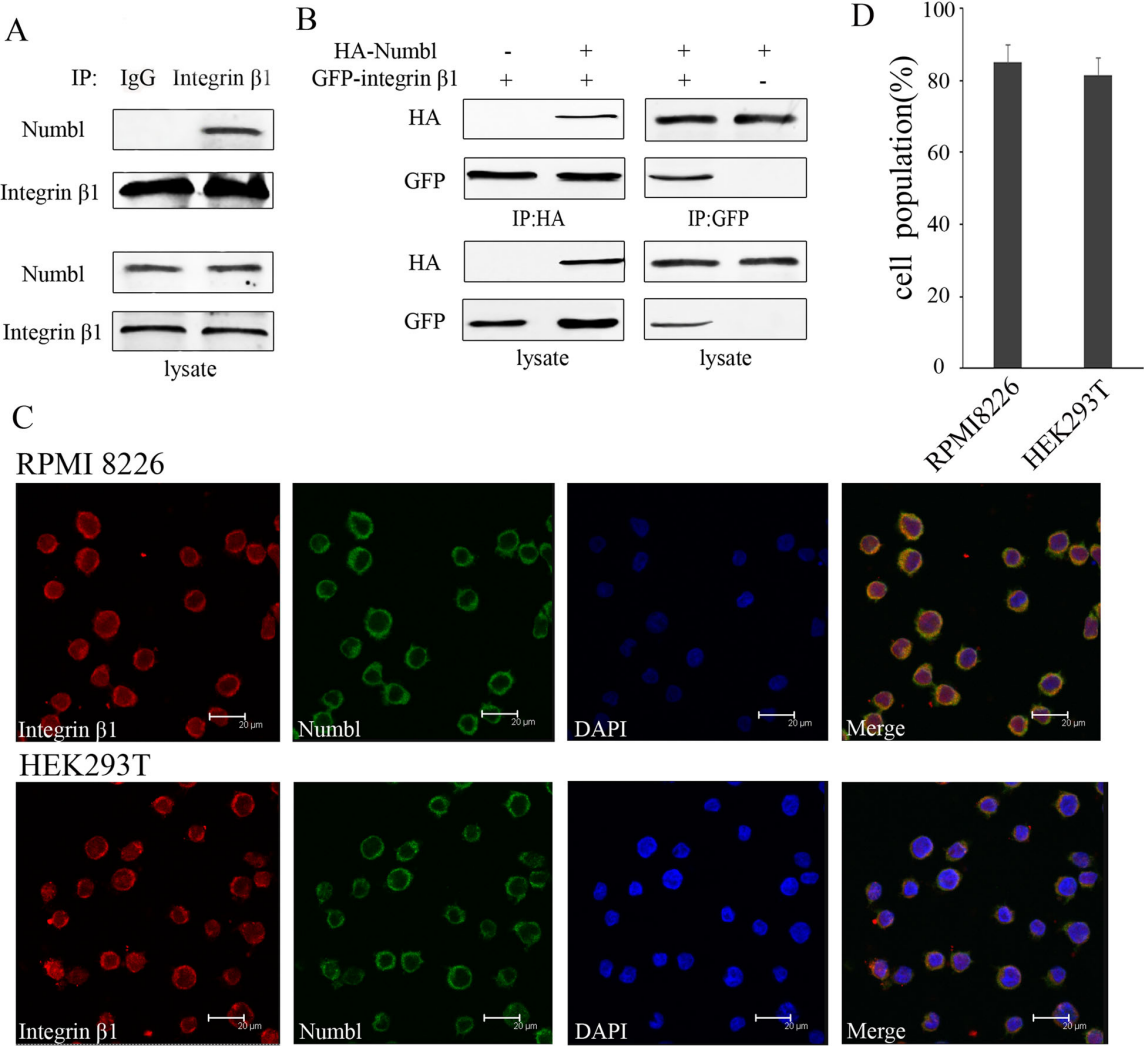
Numbl interacts with Integrin β1. **a** The interaction between endogenous Numbl and Integrin β1 in myeloma cell lysate was assessed by immunoprecipitation with an anti-Integrin β1 antibody or with a mouse normal IgG and analyzed by Western blot analysis using anti-Numbl antibody. **b** HA-tagged Numbl and GFP-tagged Integrin β1 were co-expressed in HEK293T cells. Extracts with equal amount of proteins were immunoprecipitated with anti-HA or anti-GFP antibodies and analyzed by immunoblotting with anti-GFP or anti-HA antibodies. **c** Co-localization of Integrin β1 and Numbl. The HA-Numbl and GFP-Integrin β1 plasmids were co-transfected into RPMI 8226 and HEK293T cells. After 48 h, both cells were visualized by confocal fluorescent microscopy using Hoechest 33,342 for nucleus staining. The right panel (Merge) shows the merging of all three panels (images taken with X40 magnification). **d** The quantification of images from C. A minimum of 200 cells per sample were counted, and the percentage of cells with Numbl and Integrin β1-double positive cells was calculated. Results represent the means of data from 3 independent experiments

To determine whether Numbl interacted with Integrin β1 in vivo, we performed a co-immunoprecipitation experiment. The results revealed that Numbl positively interacted with Integrin β1 (Fig. 2a). Furthermore, when HA-tagged Numbl and GFP-tagged Integrin β1 were transfected into HEK293T cells, we detected Numbl presence in the GFP-tagged Integrin β1 immunoprecipitates (Fig. 2b left). Similarly, GFP-labeled Integrin β1 was also detected in HA-tagged Numbl immunoprecipitates (Fig. 2b right). Next, we performed confocal microscopy on immunolabeled cells and showed that both Numbl and Integrin β1 are expressed in the cytoplasm, further attesting to the possibility that they may interact. These results suggest that Numbl can modulate the spatial distribution of Integrin β1, at least, in the cytoplasm (Fig. 2c).

### Domains involved in the Numbl-Intergin β1 interaction

**Fig. 3:**
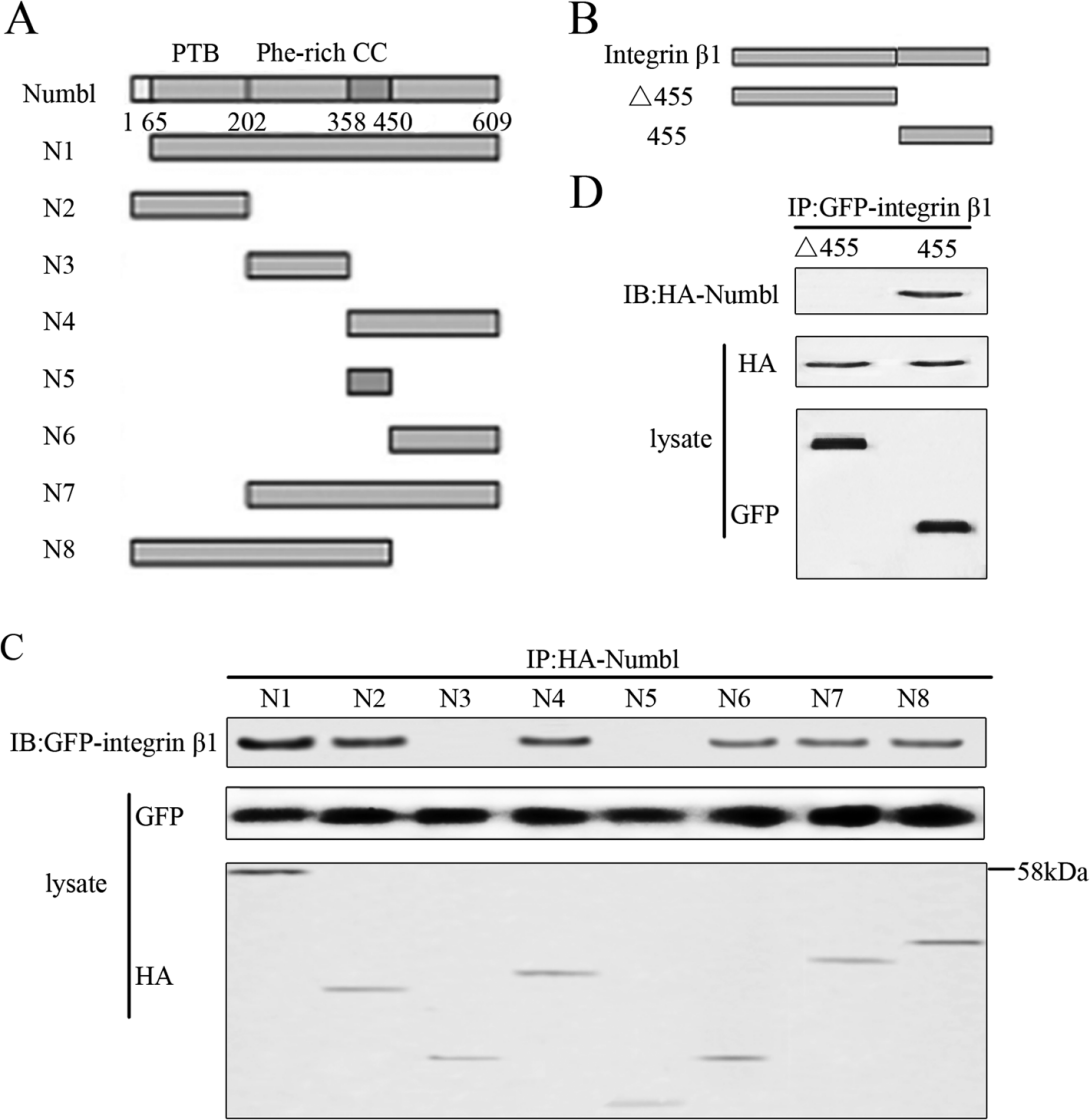
Identification of domains required for the interaction between Numbl and Integrin β1. **a** A schematic presentation of designed human Numbl derivatives. Numbl contains a phosphotyrosine binding region (PTB), a coiled-coil domain (CC), and a Phe-rich segment. **b** Schematic diagram of Integrin β1 gene and domain. **c** Two regions of Numbl are involved in its interaction with Integrin β1. HEK293T cells were co-transfected with GFP-Integrin β1 and HA-Numbl derivatives. Cell lysates were immunoprecipitated with anti-HA antibody and analyzed by Western blots with anti-GFP antibody. **d** A short N-terminal fragment (amino acids: 455–802) is required for binding with Integrin β1. HEK293T cells were transfected with the indicated expression plasmids. Immunoprecipitation and Western Blot analysis were performed using indicated antibodies

The PTB domain proteins, Numbl and Numb, have been described as essential adaptors for clathrin-mediated integrin endocytosis [25]. To further understand the association between Numbl and Intergin β1, we sought to identify which regions in these two proteins were involved in mediating their physical interaction. Numbl contains a phosphotyrosine binding region (PTB), a coiled-coil domain (CC), and a Phe-rich segment. We constructed truncation mutants of Numbl and Intergin β1 (Fig. 3a). The truncated mutants of Numbl and Intergin β1 were co-transfected into HEK293T cells, and the cell extracts were subsequently subjected to co-immunoprecipitation. Our data reveals that six Numbl mutants (N1, N2, N4, N6, N7, N8) can interact with the full-length Intergin β1 (Fig. 3c). By performing domain analysis, we found that mutants that contain PTB domain or C-terminal fragment of Numbl were capable of binding to Integrin β1. As for the Integrin β1 protein, a short N-terminal fragment (amino acid residues: 455–802), was sufficient for binding to Numbl (Fig. 3b).

### Numbl regulates the expression of integrin β1 and promotes MM cell adhesion to HS-5

**Fig. 4:**
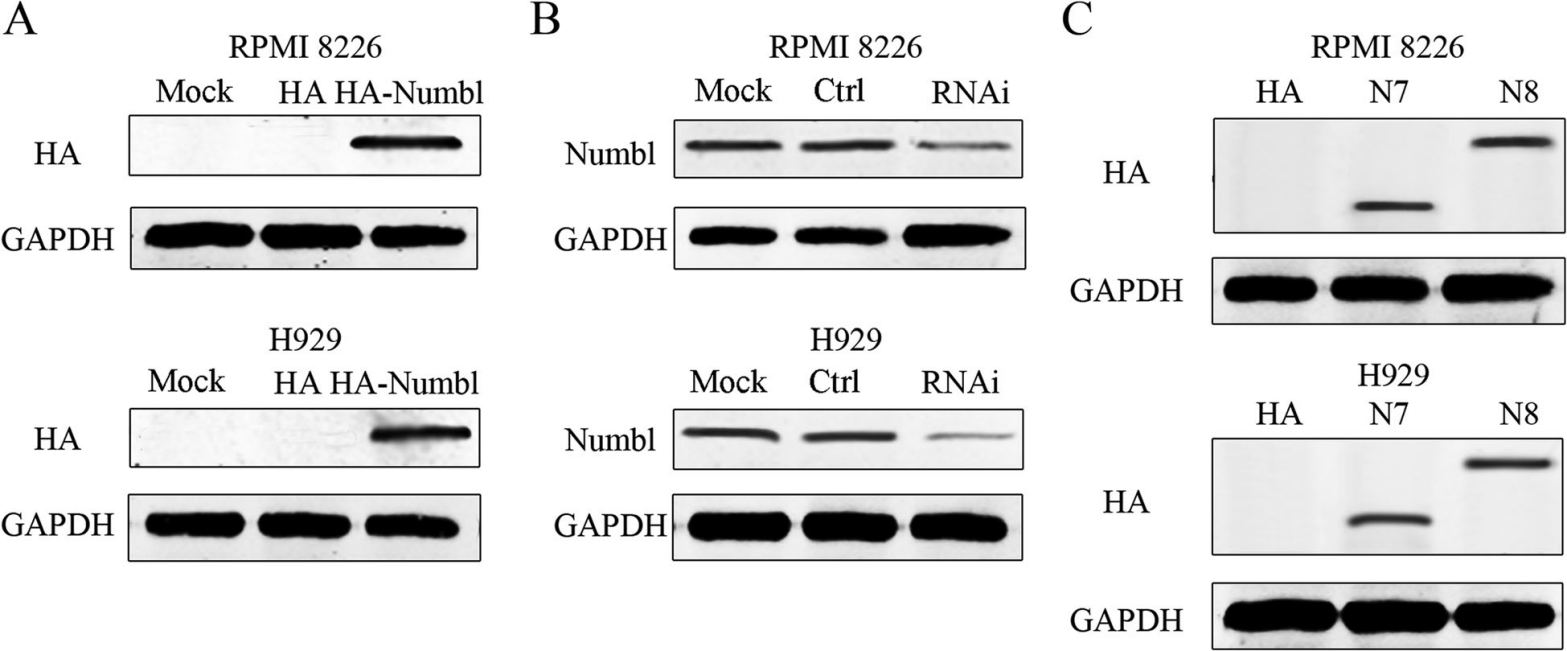
Effects of Numbl plasmids on the expression of Numbl. **a** The HA-Numbl plasmid, empty plasmid (**a**) and RNAi plasmids (**b**) or a control RNAi plasmid were transfected into RPMI 8226 and H929 cells for 48 h. Expression levels of Numbl were assessed by Western blot analysis. **c** The mutant N7 (lacking PTB domain) and N8 (lacking c-terminal domain) were transfected respectively

**Fig. 5:**
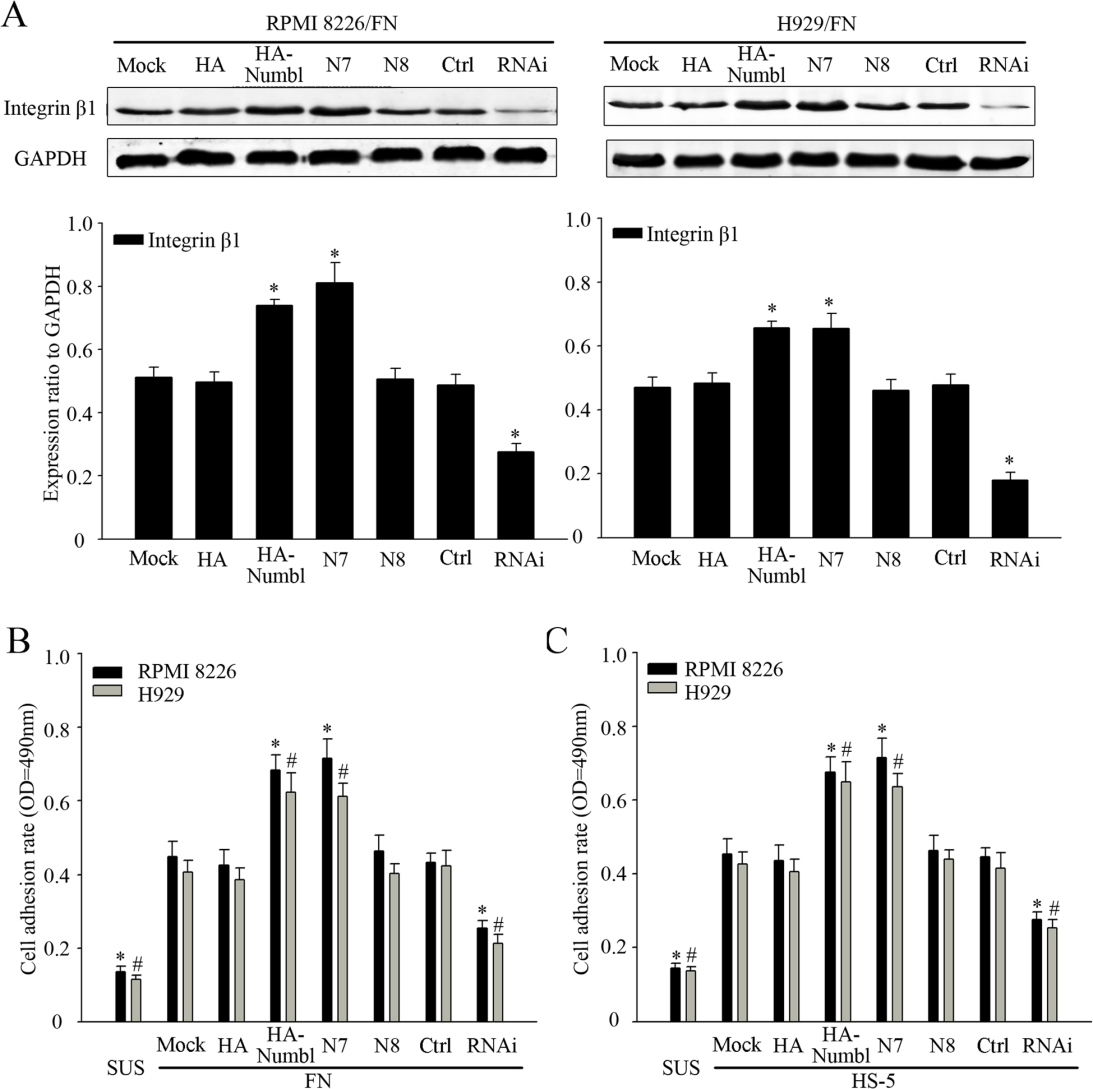
Numbl increases Integrin β1 expression and promotes myeloma cell adhesion. **a** RPMI 8226 and H929 cells were transfected with a Numbl-expressing plasmid or a control plasmid, or Numbl-specific or control siRNA plasmids for 48 h. The change in the expression of Integrin β1 was detected by Western Blot analysis (up), Western Blot results in gray value indicate quantification (down). * compared with control group (HA-Numbl compared with HA group and RNAi compared with Ctrl group, respectively), *P* < 0.05. **b**-**c** RPMI 8226 and H929 cells were transfected with Numbl-expressing plasmid or control plasmid, or Numbl-specific or control siRNA plasmids for 48 h. Cell adhesion to FN (**b**) or HS-5 (**c**) cell-coated plates was then analyzed by Calcein-AM cell adhesion assay. *, # compared with the control group (HA-Numbl compared with HA group and RNAi compared with Ctrl group, respectively), *P* < 0.05

Since Numbl was found to interact with Integrin β1, we next investigated the functional outcome of this interaction on MM cell adhesion. Full-length Numbl or RNAi were used to transfect either RPMI 8226 or H929 cell lines (Fig. 4). Furthermore, we confirmed which domains of Numb1 were responsible for the positive effect on Integrin β1 expression. Compared with full-length Numbl, overexpression of the mutant N8 (lacking C-terminal domain) did not increase Integrin β1 expression while specific knockdown of endogenous Numbl with RNAi markedly attenuated the expression of Integrin β1 (Fig. 4a). We also detected the effect of mutants N3 and N5 that did not bind to Integrin β1. Negither of the mutants worked on Integrin β1(Additional file 1: Figure S1A). These results confirm that Numbl regulates the expression of Integrin β1 through the C-terminal fragment rather than the PTB domain. Furthermore, we questioned whether overexpression of Numbl influences myeloma cell adherence to HS-5 cells or to FN. To this end, we used Calcein-AM test to assess cell adhesion in 96-cell plates and a microplate reader was used for measurement. As was shown in Fig. 5b and c, overexpression of full-length Numbl and N7(△PTB) caused more cells to adhere to HS-5 cells and FN, whereas overexpression of N8 did not affect adherence. So do N3 and N5 (Additional file 1: Figure S1B). Moreover, knockdown of endogenous Numbl reduced further myeloma cell adhesion compared to control group. Since mutant N8 could bind to Integrin β1 without impacting the protein levels, we decided to use N8 as the dominant negative mutant of full-length Numbl in the ensuing experiments.

### Numbl promotes the drug resistance in MM cells

**Fig. 6:**
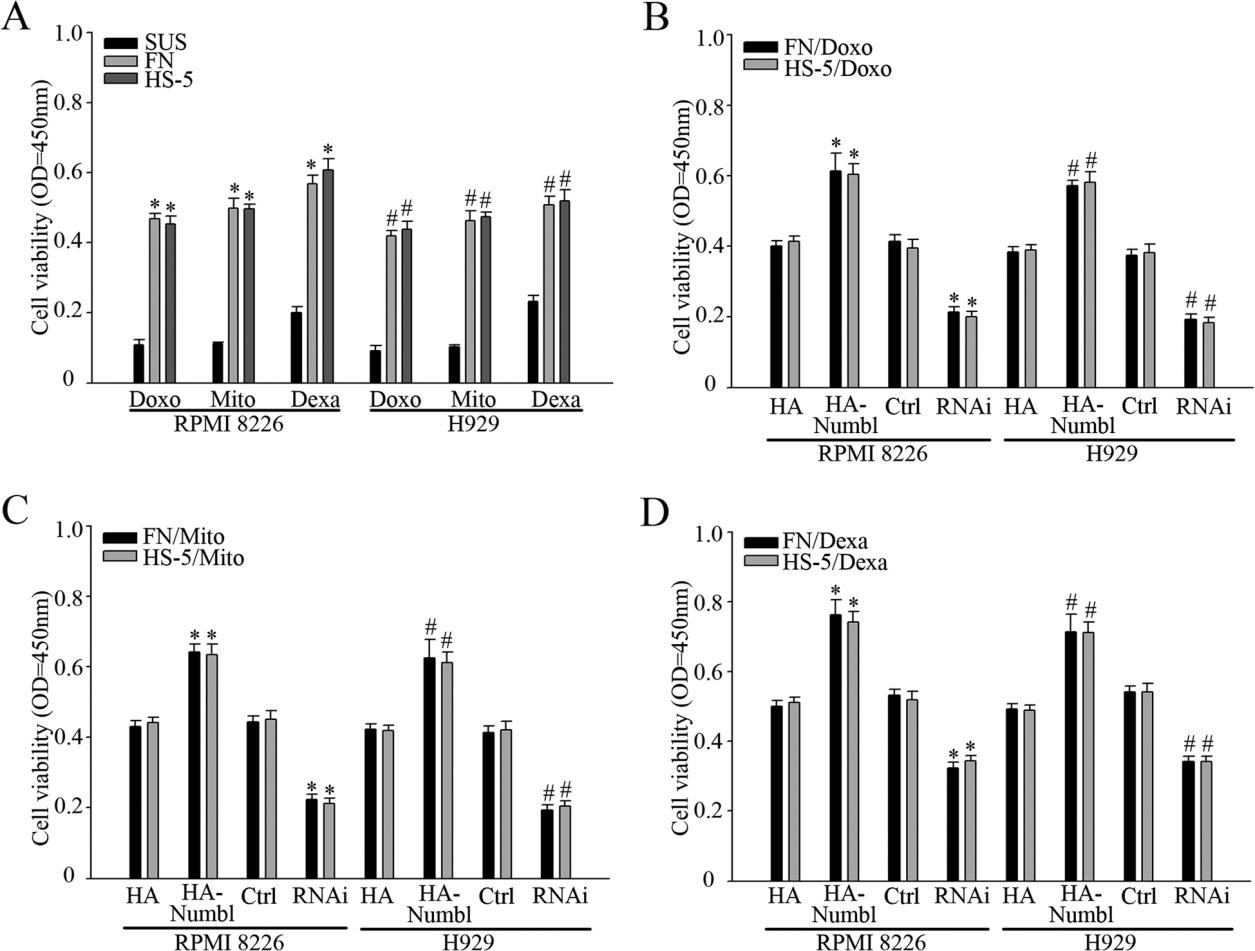
The positive regulation of CAM-DR in myeloma cell lines by Numbl. **a** Myeloma cell activity in suspension and in adhesion model after treatment with different drugs. *, # compared to suspension group (SUS), *P* < 0.05. **b**-**d** In the adhesion model of myeloma cells, myeloma cell activity was detected after alteration of the Numbl expression and treatment with 1 μM doxorubicin (**b**), 2 μM mitoxantrone (**c**) and 0.05 μM dexamethasone (**d**) for 72 h. *, # compared with the control group (HA-Numbl compared with HA group and RNAi compared with Ctrl group, respectively), *P* < 0.05

**Fig. 7:**
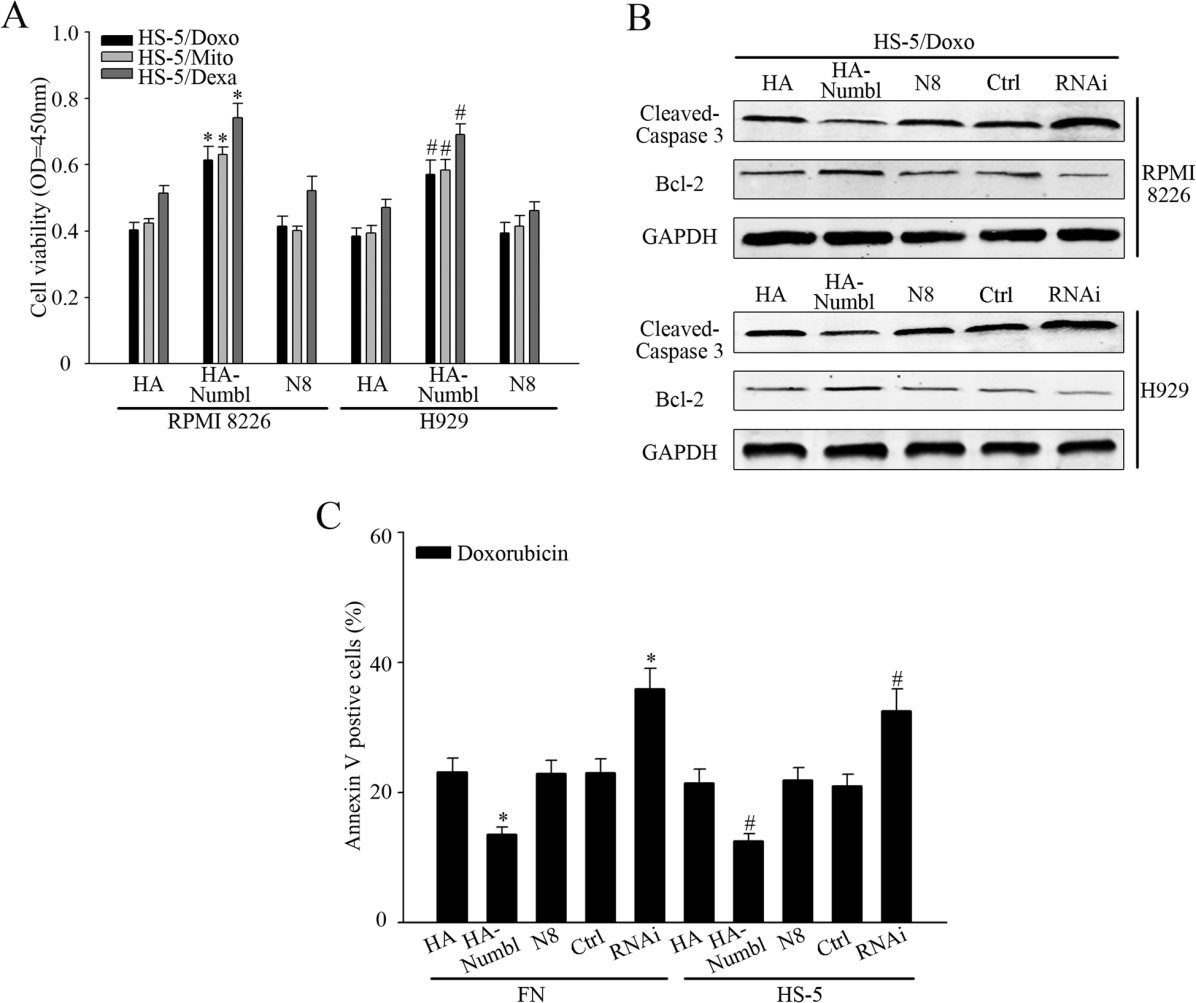
Numbl regulates CAM-DR process in myeloma cells through interaction with Integrin β1. **a** In the adhesion model of myeloma cells, after alterating the interaction between Numbl and Integrin β1, myeloma cell activity with different drugs was assessed. *,# compared with the control group, *P* < 0.05. **b** After the alteration of the Numbl expression and the interaction between Numbl and Integrin β1, we probed for the expression of Caspase-3 and Bcl-2 in myeloma cell adhesion-mediated drug resistance model through Western Blot analysis. **c** Flow cytometry was used to measure the cell apoptosis in a cell adhesion-mediated drug resistance model. *, # compared with the control group (HA-Numbl compared with HA group and RNAi compared with Ctrl group, respectively), *P* < 0.05

The observation that Numbl promotes adhesion of MM cells to FN and also physically associates with Integrin β1 led us to examine whether Numbl could enhance CAM-DR development observed in MM cells. To test this hypothesis, RPMI 8226 and H929 cells were treated with Doxorubicin (Dox), mitoxantrone (Mito) and dexamethasone (Dexa) in the presence and absence of HS-5 cells or FN. As expected, when RPMI 8226 and H929 cell adhere to FN or HS-5 cells, MM cell viability was increased with the addition of chemotherapeutic drugs (Fig. 6a). Furthermore, to detect whether Numbl plays a role in the progress of CAM-DR in cells, RPMI 8226 and H929 cells, with altered Numbl expression levels, were treated with Dox, Mito, Dexa respectively, as indicated above. While overexpression of Numbl significantly protected MM cell from Dox-induced cell death in HS-5 cells or FN coated plates, suppressing the intrinsic expression of Numbl using specific siRNAs enhanced MM cell death upon Dox treatment (Fig. 6b). Similarly, Numbl overexpression promoted MM cell survival after treatment with Mito or Dexa(Fig. 6c and d). To assess whether Numbl promoted the survival of chemotherapeutics-treated MM cells via Integrin β1, we studied the role of mutant N8 (with absent C-terminal domain). As shown in Fig. 7a, overexpression of N8 by MM cells could not increase their viability after treatment with chemotherapeutic drugs. Overexpression of N3 and N5 that did not bind to Integrin β1 also couldn’t protect cells against chemotherapeutic drugs (Additional file 1: Figure S1C).

To test directly whether Numbl-induced drug resistance of MM cells was due to inhibition of apoptosis, MM cells overexpressing Numbl were treated with Dox in the presence of HS-5 cells, before being harvested for WB and Annexin V analysis. Compared with the vector control and mutant N8 transfection, full-length Numbl transfected cells had significantly lower expression levels of anti-apoptotic protein, BCL2, and lower cell apoptosis in the presence of HS-5 cells. While mutant N3 did not act on myeloma cell apoptosis (Additional file 1: Figure S1D). In contrast, silencing of Numbl markedly raised the expression of cleaved-caspase 3 and the number of Annextin V positive cells (Fig. 7c). Taken together, these results indicate that Numbl specifically promotes MM cell drug-resistance, likely through the alteration of Integrin β1 expression, leading to inhibition of apoptosis.

### Numbl affect cell cycle through the interaction with integrin β1

**Fig. 8:**
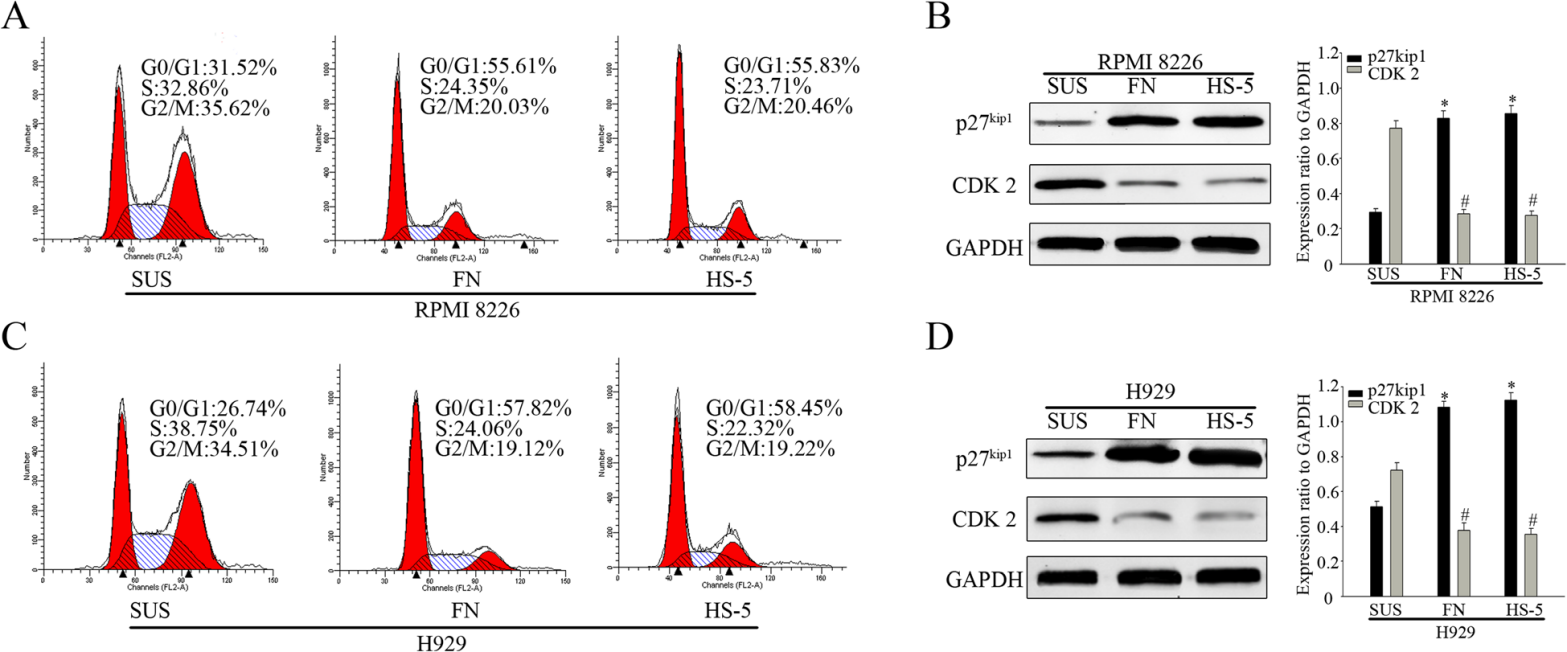
The influence of cell adhesion on the cell cycle of myeloma. **a** Cell cycle stage was determined by flow cytometry in the myeloma cell line RPMI8226 in both suspension and in adhesion models. **b** The expression of cell cycle proteins were analyzed by Western Blot analysis (from A) (left) and the results of gray value quantification (right). **c** Cell cycle detection by flow cytometry in myeloma cell line H929 in both suspension and in adhesion models. **d** The expression of cell cycle proteins was analyzed by Western Blot analysis (from C) (left) and the results of gray value quantification (right). *, # compared to suspension group (SUS), *P* < 0.05

**Fig. 9:**
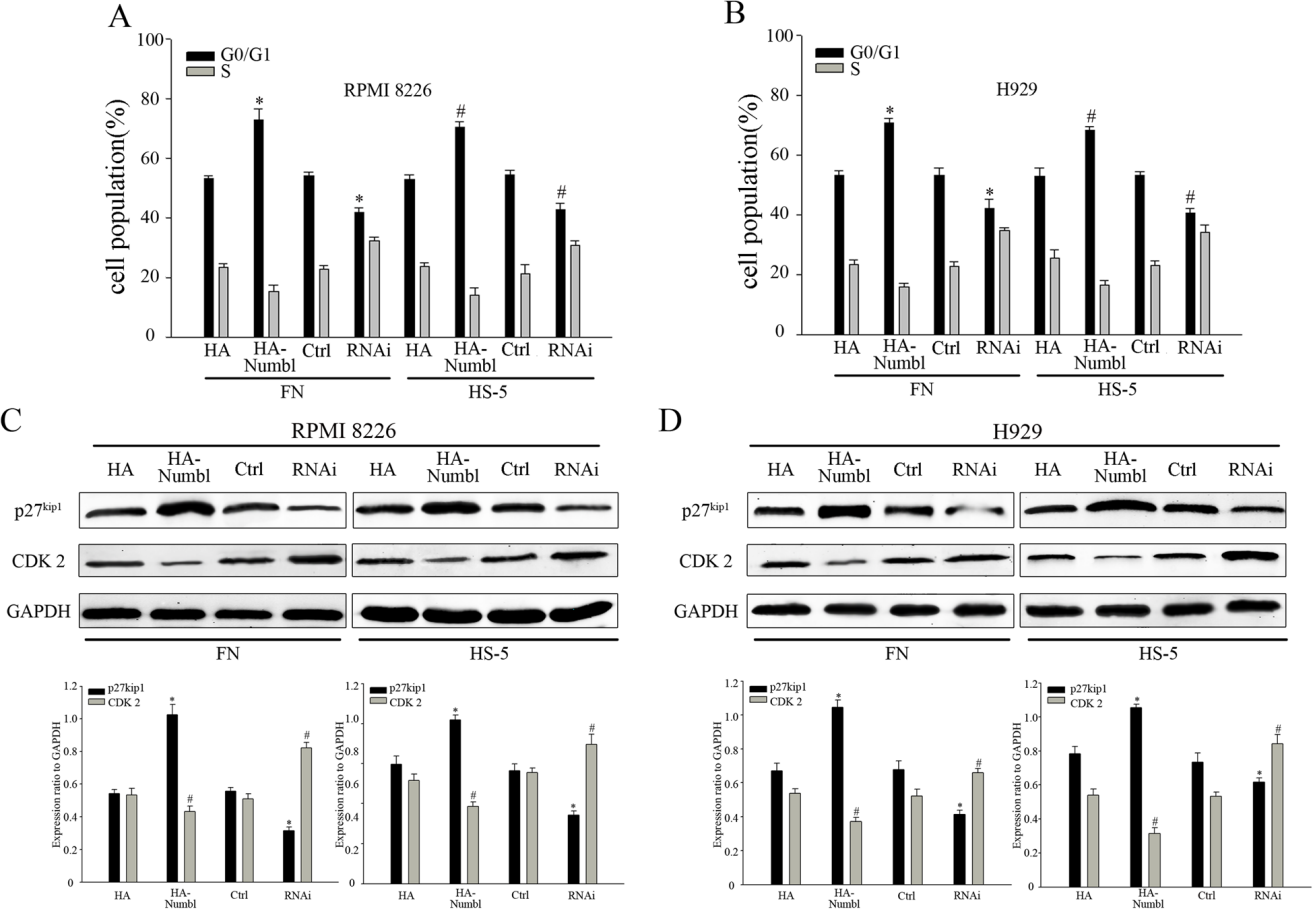
Numbl affects myeloma cell cycle. **a**-**b** After Alteration of Numbl expression in the adhesion model of myeloma cell lines, RPMI 8226 (**a**) and H929 (**b**), the cell cycle was analyzed by flow cytometry *, # compared with the control group (HA-Numbl compared with HA group and RNAi compared with Ctrl group, respectively), *P* < 0.05. **c**-**d** The expression of cell cycle proteins assessed by Western Blot (From A-B) (up) and the results of gray value quantification (down). *, # compared with the control group (HA-Numbl compared with HA group and RNAi compared with Ctrl group, respectively), *P* < 0.05

**Fig. 10:**
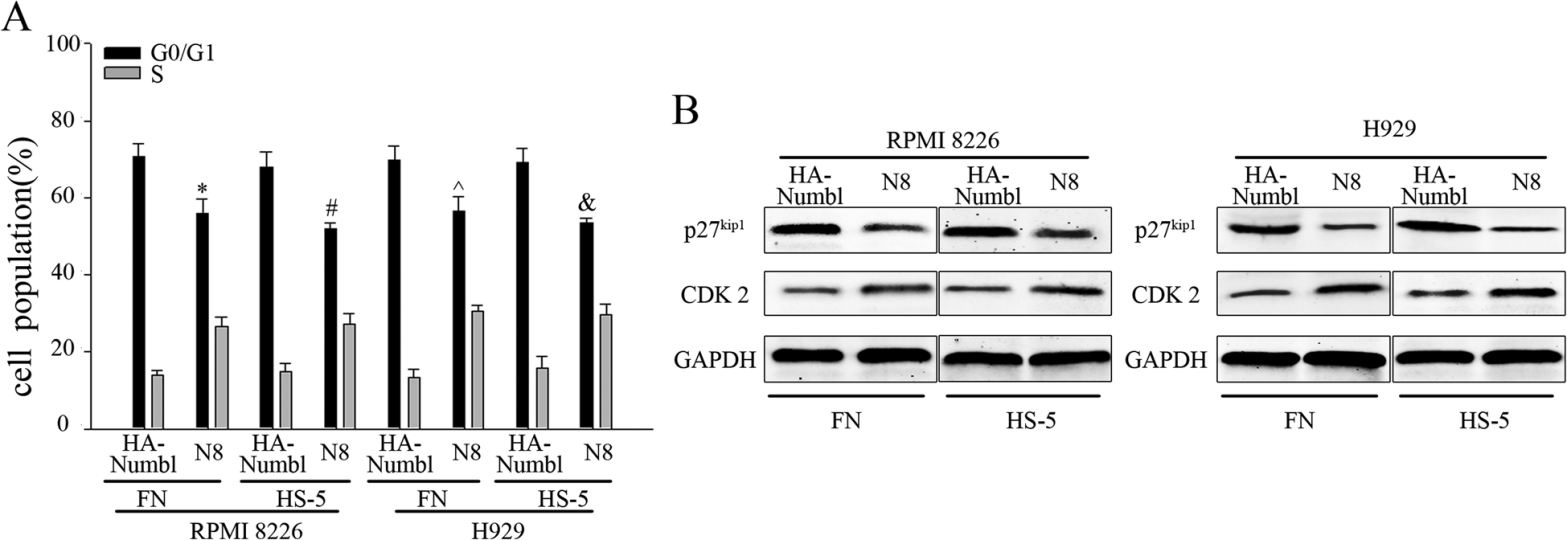
Numbl modulates myeloma cell cycle by interacting with the Integrin β1. **a** In the adhesion model of myeloma cells, the interaction between Numbl and Integrin β1 was altered and the cell cycle was analyzed by flow cytometry. *, mutant N8 compared with HA-Numbl group in the presence of FN with RPMI 8226 cell; #, mutant N8 compared with HA-Numbl group in the presence of HS-5 with RPMI 8226 cell; ^, mutant N8 compared with HA-Numbl group in the presence of FN with H929 cell; &, mutant N8 compared with HA-Numbl group in the presence of HS-5 cells with H929 cell, *P* < 0.05. **b** Detection of cell cycle proteins by WB (from **a**)

We were interested to further define the mechanisms by which Numbl contributes to the CAM-DR induction. Previous studies demonstrated that cell adhesion to FN may induce cell cycle arrest and protect tumor cells from chemotherapeutic drug-induced apoptosis [2, 5, 7]. First, we analyzed the cell cycle distribution of MM cells in suspension and in adhesion conditions. Flow cytometric analysis showed that MM cells adhering to FN or HS-5 cells remained in G0/G1 phase. (Fig. 8a, c). Consistently, we detected the expression of proteins that regulate the cell cycle progression in G0/G1 restriction point. As expected, a significantly elevated level of p27^kip1^ was detected in adherent cells, whereas CDK2 was downregulated (Fig. 8b and d). Next, we sought to determine whether Numbl expression could affect the cell adhesion-mediated cell cycle arrest. As shown in Fig. 9a and b, flow cytometric analysis revealed that overexpression of Numbl triggered FN-adherent MM cells to be arrested in the G0/G1 phase (72.7% of the cells). In comparison HA group resulted in only 53.2% of cells arrested in the G0/G1 phase. And the effect of N3 group was similar with HA group(Additional file 1: Figure S1E). Complete suppression of Numbl led to even less cells arrested in the G0/G1 phase (41.8%) compared to the Ctrl group (54.1%). Cell cycle related proteins p27^kip1^ and CDK2 expression were consistent with the above data trend (Fig. 9c, d). Given these data, we propose that Numbl might be involved in cell cycle regulation through an interaction with Integrin β1. Indeed, a comparison of the effect of the full length Numbl and the mutant Numbl (N8) on cell cycle regulation showed that mutant N8 had no effect on cell cycle distribution (56.1%) and was comparable to control. However, the full length Numbl achieved 70.8% of cell arrest in the G0/G1 phase (Fig. 10a). These data were confirmed with Western Blot analysis (Fig. 10b).

### Numbl regulates activation of signaling pathways through interaction with integrin β1

**Fig. 11:**
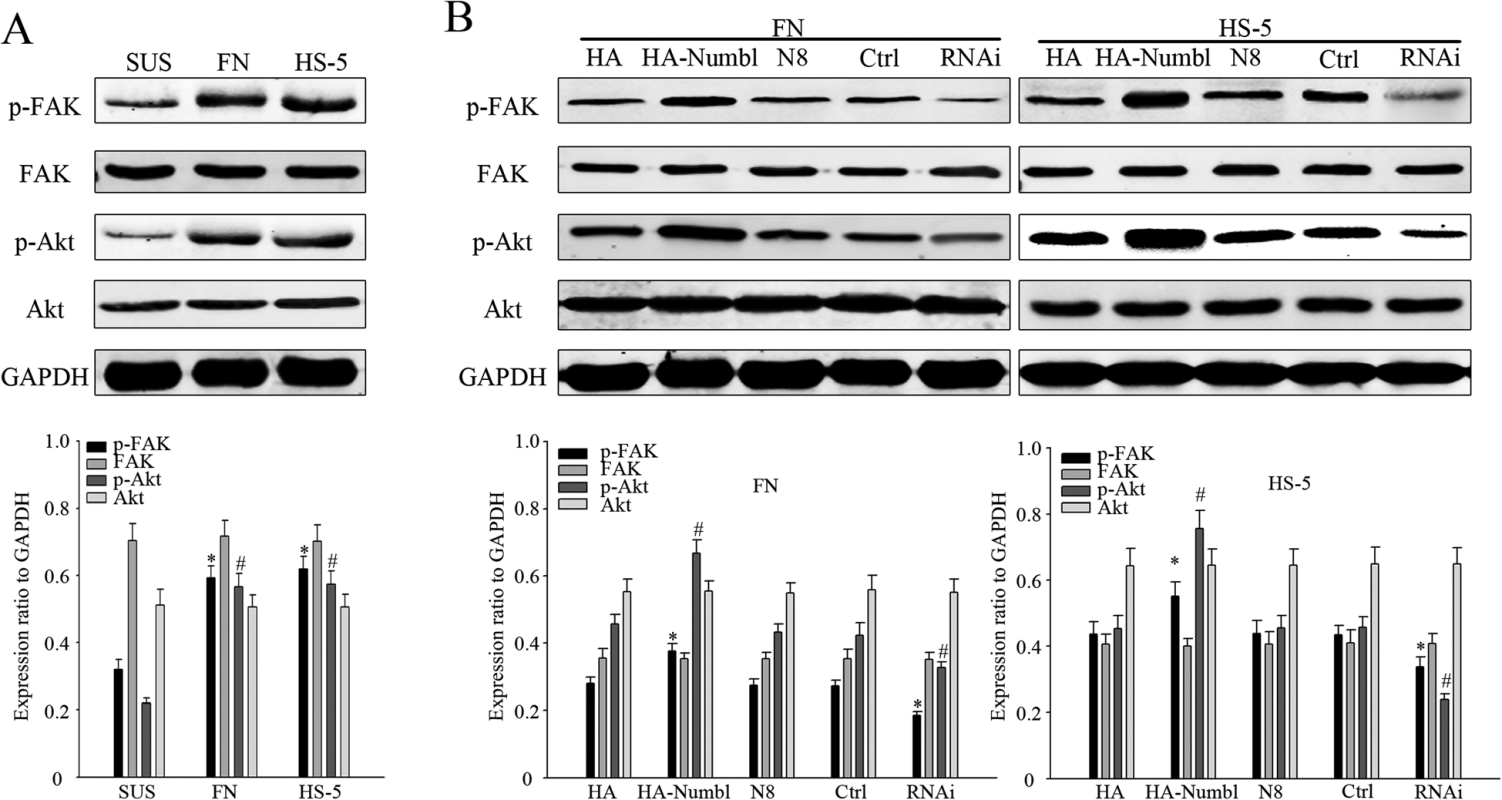
Cell adhesion influences the activation of the FAK and PI3K/AKT signaling pathway. **a** Western Blot analysis revealed activation of FAK and the PI3K/AKT signaling pathway in myeloma cells in both suspension and in adhesion models (up) and the results of gray value quantization (down). *, # compared to suspension group (SUS), *P* < 0.05. **b** Western Blot analysis revealed activation of FAK, PI3K/AKT signaling pathway in myeloma cells after intervention Numbl expression in cell adhesion model (up) and the results of gray value quantization (down). *, # compared with the control group (HA-Numbl compared with HA group and RNAi compared with Ctrl group, respectively), *P* < 0.05

**Fig. 12:**
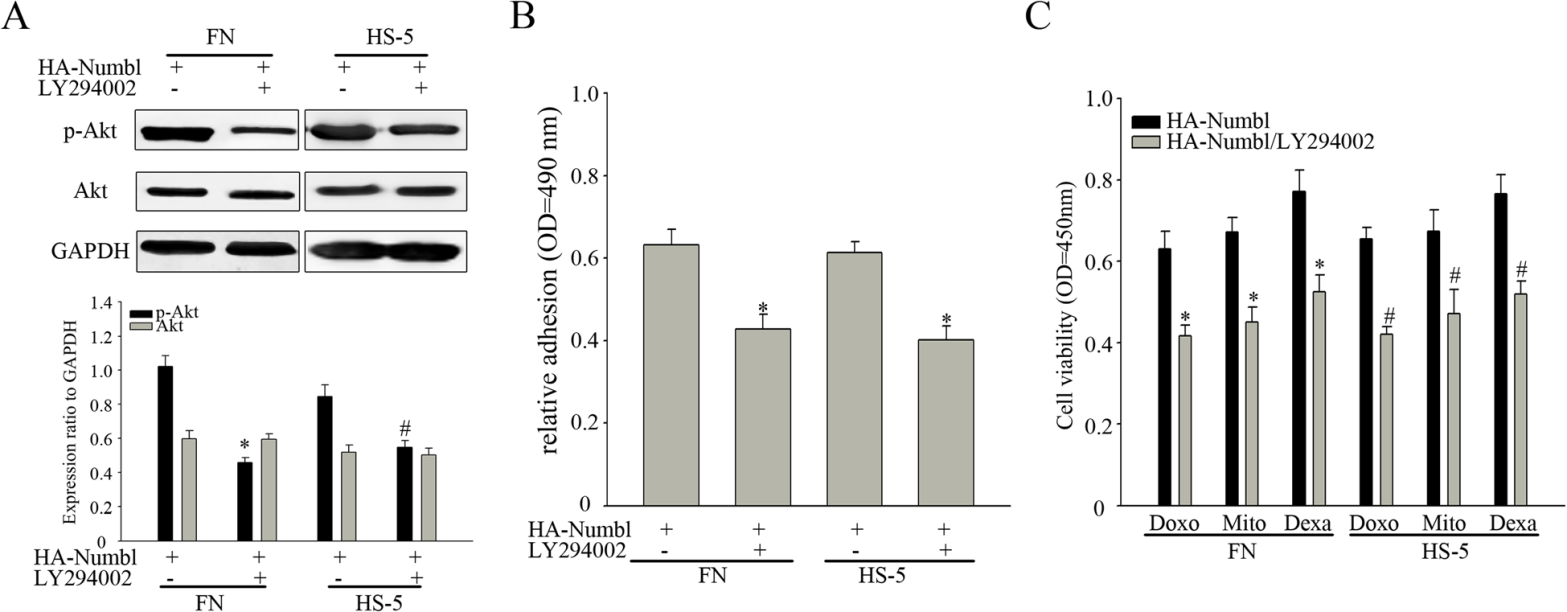
Numbl regulates the activation of the PI3K/AKT signaling pathway. **a** Myeloma adhesion model overexpressing Numbl were treated with LY294002 and the PI3K/AKT signaling pathway activation state (up) was assessed by WB, and the results of gray value quantization (down). *, # compared with the control group, *P* < 0.05. **b** Myeloma adhesion model overexpressing Numbl were treated with LY294002 and cell adhesion rate was analyzed using Calcein-AM, * compared with the control group, *P* < 0.05. **c** Myeloma adhesion model overexpressing Numbl were treated with LY294002 and different chemotherapy drugs, cell activity was evaluated. *, # compared with the control group, *P* < 0.05

FAK/Src has been shown to activate phosphoinositide 3-kinase (PI3K) and Akt has been shown to promote cell adhesion and survival [40, 41]. As such, the activation of FAK and the PI3K/AKT signaling pathways were examined in the MM cell adhesion model. The results showed that these signaling pathways were indeed activated (Fig. 11a). By altering the expression of Numbl, we showed that Numbl could regulate the activation of these signaling pathways (Fig. 11b). In order to further test the hypothesis that high expression of Numbl could influence the activation of signaling pathways, we treated MM cells with a PI3K inhibitor (LY294002) after Numbl overexpression. Treatment with LY294002 reversed the enhanced activity of the cell signaling pathways (Fig. 12a). In addition, treatment with LY294002 reversed the increase in the cell adhesion rate and the drug resistance conferred by the high expression of Numbl (Fig. 12b, c). In conclusion, Numbl triggers the activation of cell signaling pathways by regulating Integrin β1 in a way to impact the progress of MM cell adhesion-mediated drug resistance.

## Discussion

MM, also known as myeloma, plasma cell myeloma or Kahler’s disease, is a progressive malignant tumor. It is mainly characterized by tumor cell accumulation in the bone marrow and secretion of monoclonal immunoglobulins [42]. Although new treatments are continuously being developed, MM remains incurable due to a high recurrence rate, drug resistance following initial treatment, and low sensitivity to chemotherapy and radiotherapy [43, 44]. The average survival time of patients with MM after relapse is about 9 months and resistance to drug is the main obstacle to a consistently effectiӱignalingetn [4, 45]. The relationship between MM cells and bone marrow is a complex network. Bone marrow provides a micro-environment for MM cells to survive in the presence of the disease by mean of a soluble stromal connecting MM cells and mediating the interactions [46]. This also suggests that MM cell adhesion to bone marrow microenvironment or extracellular matrix can induce the resistance to multiple drug-mediated apoptosis, a process known as CAM-DR. [6, 45, 47]. Cell adhesion plays a pivotal role in the process of the resistance to drug mediated apoptosis [48]. Therefore, herein, we describe the development of an in vitro adhesion model simulating in vivo MM cell adhesion to study the role and mechanism of adhesion in mediating CAM-DR.

Numb aa sequence homology to Numbl is high and previous studies have showed that Numbl plays redundant roles with Numb in the regulation of cellular differentiation, proliferation, and fate decision [49–52]. It has been reported that Numb interacts with Integrin β1 through its PTB domain and it affects cell adhesion by controlling the internalization and compartmentalization of integrin β1 [23]. Therefore, here we sought to investigate whether Numbl interacts in the same way with Integrin β1 in myeloma cells. In this experiment, we detected the expression levels of Numbl and Integrin β1 when MM cells are co-cultured with either FN or HS-5 stromal cells. Our data revealed that Numbl expression levels were significantly higher in co-cultures than in MM cells cultured alone. This is suggestive of the possibility that Numbl plays a role in CAM-DR development in MM. Subsequently, we confirmed the occurrence of an interaction between endogenous or exogenous Numbl and Integrin β1 in the cells, using CO-IP and immunofluorescent staining assays. In order to further characterize this interaction, we designed truncated mutation plasmid constructs of Numbl and Integrin β1. Domain mapping experiments indicated that PTB domain and C-terminal of Numbl are responsible for binding to Integrin β1. Indeed, only the expression of full-length and N7 (lacking PTB domain) mutant versions could increase the protein levels Integrin β1, whereas expression of N8 (lacking C-terminal domain) mutant had no effect. We hypothesized that the C-terminal domain of Numbl assumes a specific conformation that facilitates Integrin β1 binding, while PTB domain cannot. Accordingly, we advanced the idea that the interaction between C-terminal domain of Numbl and Integrin β1 might be highly important for the development of MM CAM-DR. To test this, we altered Numbl expression and investigated the role of its full length and N8 (C-terminal absence) fragment in adherent myeloma cell with or without chemotherapeutic drug. We have also developed and identified additional truncation mutants of Numbl (N3 and N5) that showed interference with the interaction (Additional file 1: Figure S1). Overall, our data indicates that Numbl promotes drug resistance of MM cells by means of an interaction between the C-terminal domain of Numbl and Integrin β1. Here we reported about the expression and the role of Numbl in the progress of MM CAM-DR, however the specific regulation mechanism is yet to be elucidated and warrant further investigations.

Most of the cytotoxic effects of chemotherapy drugs on tumor cells are mediated through targeting a specific cell cycle stage. When tumor cells adhere to extracellular matrix, cell cycle is delayed and cells are in a relatively quiescent state, raising the cell resistance to chemotherapeutic drugs [7, 26, 27, 48]. Proteins regulating cell cycle play a key role in the cell cycle progression [28]. We had demonstrated that over-expression of Numbl induced cell cycle arrest in FN-adherent MM cells, whereas, N8 mutant did not. Taken together, these results suggest that Numbl is a negative regulator of cell cycle progression, in part, via association with Integrin β1-mediated adhesion. It has been reported that Integrin β1 cannot only affect the cell cycle, but it can also activate the downstream corresponding signaling pathways, including FAK and PI3K/AKT pathways, while also mediating cell adhesion. It has been previously reported that phosphorylation of the Focal Adhesion Kinase (FAK) led to the activation of Src/Syk/STAT3 and Akt, which are crucial signaling molecules involved in enhancing cell adhesion and protecting cells from drug-induced cell apoptosis [53]. Activating Integrin β1/FAK and its downstream Src-Syk-STAT3/Akt signaling pathway can promote enhanced cell adhesion and CAM-DR in MM cells [53, 54]. In this study, we have shown that overexpression of Numbl promoted the adhesion of myeloma cells to FN or HS-5 cells via activation of Integrin β1-FAK and the subsequent activation of Akt pathway, which eventually facilitated the survival and drug resistance of myeloma cells. Indeed, the treatment of MM cells with LY294002, an inhibitor of phosphoinositide 3-kinase, decreased the activation of Akt, along with cell adhesion and the cell survival rates after treatment with chemotherapy drugs. Taken together, these results indicate that Numbl promotes the adhesion and CAM-DR of MM cells by activating Integrin β1/FAK and its downstream Akt pathway.

Overall, we have demonstrated that Numbl promotes MM cell adhesion, survival, and drug resistance via its C-terminal domain binding to Integrin β1. Subsequently, integrin β1 activation leads to FAK phosphorylation, which in turn activates Akt pathway, leading to the promotion of CAM-DR in MM cells. Our findings further highlight the therapeutic potential of targeting the Numbl/Integrin/FAK/Akt axis.

## Conclusions

In a myeloma adhesion model, the expression levels of Numbl and Integrinβ1 were significantly increased. Numbl affects CAM-DR through regulating Integrin β1 activation, which affects the cell cycle and activation of downstream signaling pathway kinase.

## Supplementary information

**Additional file 1: Figure S1.** (A) RPMI 8226 and H929 cells were transfected with either a Numbl-expressing plasmid or Numbl-specific plasmid for 48 h and the relative preponderence of the Integrin β1 lelves were detected by Western Blot. (B) Cell adhesion to HS-5 cell-coated plates was then analyzed by Calcein-AM cell adhesion assay. *, HA-Numbl compared with HA group, *P* < 0.05. (C) In the adhesion model of myeloma cells, after the intervention of the interaction between Numbl and Integrin β1, myeloma cell activity with different drugs was detected. *, # HA-Numbl compared with HA group, *P*< 0.05. (D) Flow cytometry measured the cell apoptosis in cell adhesion mediated drug resistance model (Left). *, # HA-sNumbl compared with HA group, *P*< 0.05. After the intervention of the Numbl expression and the interaction between Numbl and Integrin β1, Caspase-3 and Bcl-2 expression in RPMI 8226 cell adhesion mediated drug resistance model by Western Blot (Right). (E) In the adhesion model of RPMI 8226 cells, the interaction between Numbl and Integrin β1 was altered and cell cycle stage was analyzed by flow cytometry (Left). *, HA-Numbl compared with HA group, *P*< 0.05. Detection of cell cycle proteins by WB (Right).

## Data Availability

All data supporting the conclusions have been presented in this manuscript. The corresponding author shall provide the raw data and plasmids as per request.

## Abbreviations

CAM-DR: Cell adhesion mediated drug resistance
CC: Coiled-coil domain
CCK8: Cell Counting Kit-8
CO-IP: Co-immunoprecipitation
Dexa: Dexamethasone
Dox: Doxorubicin
ECL: Enhanced chemiluminescent
EM-DR: Microenvironment mediated resistance
FAK: Focal Adhesion Kinase
FN: Fibronectin
HEPES: N-2-hydroxyethylpiperazine-N-ethane-sulphonicacid
MCL: Mantle cell lymphoma
Mito: Mitoxantrone
MM: Mutiple myeloma
NEU3: Plasmalemma neuraminidase
PI3K: Phosphatidylinositol 3-kinase
PKB: Protein kinase B
PTB: Phosphotyrosine binding
PVDF: Polyvinylidene Fluoride
SDS–PAGE: Sodium dodecyl sulfate–polyacrylamide gel electrophoresis
SFM-DR: Soluble cytokines mediated drug resistance
