# Supplementary figures and images for: Elucidating the expression and function of Numbl during cell adhesion-mediated drug resistance (CAM-DR) in multiple myeloma (MM)
Source: BMC Cancer. 2019 Dec 30;19:1269. doi: 10.1186/s12885-019-6446-y (PMC6937660; doi:10.1186/s12885-019-6446-y)

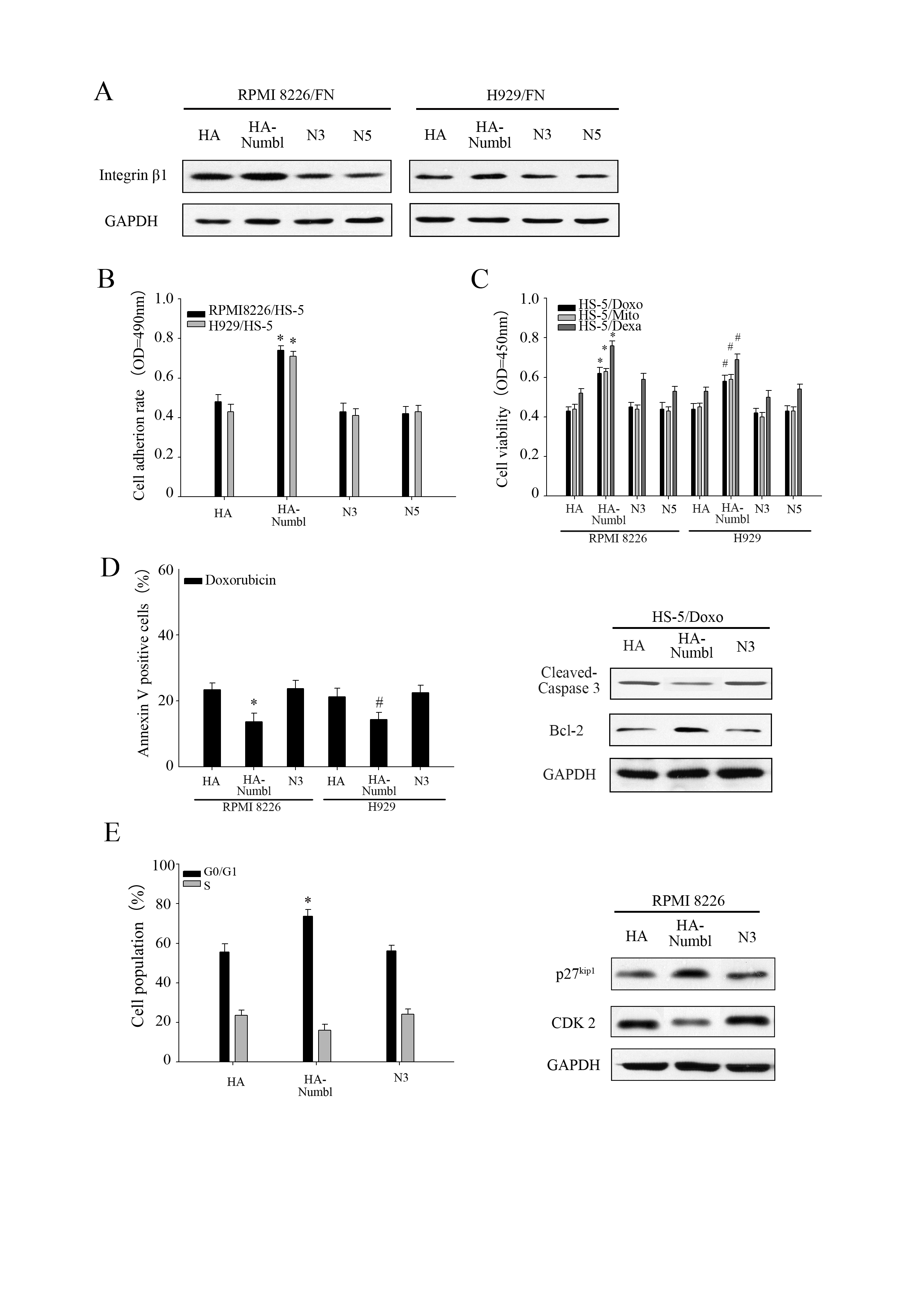

Supplement: Supplementary file 1 — Additional file 1: Figure S1. (A) RPMI 8226 and H929 cells were transfected with either a Numbl-expressing plasmid or Numbl-specific plasmid for 48 h and the relative preponderence of the Integrin β1 lelves were detected by Western Blot. (B) Cell adhesion to HS-5 cell-coated plates was then analyzed by Calcein-AM cell adhesion assay. *, HA-Numbl compared with HA group, P < 0.05. (C) In the adhesion model of myeloma cells, after the intervention of the interaction between Numbl and Integrin β1, myeloma cell activity with different drugs was detected. *, # HA-Numbl compared with HA group, P< 0.05. (D) Flow cytometry measured the cell apoptosis in cell adhesion mediated drug resistance model (Left). *, # HA-sNumbl compared with HA group, P< 0.05. After the intervention of the Numbl expression and the interaction between Numbl and Integrin β1, Caspase-3 and Bcl-2 expression in RPMI 8226 cell adhesion mediated drug resistance model by Western Blot (Right). (E) In the adhesion model of RPMI 8226 cells, the interaction between Numbl and Integrin β1 was altered and cell cycle stage was analyzed by flow cytometry (Left). *, HA-Numbl compared with HA group, P< 0.05. Detection of cell cycle proteins by WB (Right). [file 12885_2019_6446_MOESM1_ESM.tif]
